# Insights into the Genomic Architecture of Seed and Pod Quality Traits in the U.S. Peanut Mini-Core Diversity Panel

**DOI:** 10.3390/plants11070837

**Published:** 2022-03-22

**Authors:** Jinesh D. Patel, Ming Li Wang, Phat Dang, Chris Butts, Marshall Lamb, Charles Y. Chen

**Affiliations:** 1 Department of Crop, Soil and Environmental Sciences, Auburn University, Auburn, AL 36849, USA; jdp0078@auburn.edu; 2 USDA-ARS Plant Genetic Resources Conservation, Griffin, GA 30223, USA; mingli.wang@usda.gov; 3 USDA-ARS National Peanut Research Laboratory, Dawson, GA 39842, USA; phat.dang@usda.gov (P.D.); chris.butts@usda.gov (C.B.); marshall.lamb@usda.gov (M.L.)

**Keywords:** diversity panel, GWAS, seed quality traits, peanut

## Abstract

Traits such as seed weight, shelling percent, percent sound mature kernels, and seed dormancy determines the quality of peanut seed. Few QTL (quantitative trait loci) studies using biparental mapping populations have identified QTL for seed dormancy and seed grade traits. Here, we report a genome-wide association study (GWAS) to detect marker–trait associations for seed germination, dormancy, and seed grading traits in peanut. A total of 120 accessions from the U.S. peanut mini-core collection were evaluated for seed quality traits and genotyped using Axiom SNP (single nucleotide polymorphism) array for peanut. We observed significant variation in seed quality traits in different accessions and different botanical varieties. Through GWAS, we were able to identify multiple regions associated with sound mature kernels, seed weight, shelling percent, seed germination, and dormancy. Some of the genomic regions that were SNP associated with these traits aligned with previously known QTLs. For instance, QTL for seed dormancy has been reported on chromosome A05, and we also found SNP on the same chromosome associated with seed dormancy, explaining around 20% of phenotypic variation. In addition, we found novel genomic regions associated with seed grading, seed germination, and dormancy traits. SNP markers associated with seed quality and dormancy identified here can accelerate the selection process. Further, exploring the function of candidate genes identified in the vicinity of the associated marker will assist in understanding the complex genetic network that governs seed quality.

## 1. Introduction

The genus of *Arachis* contains 30 diploid wild species, a wild tetraploid species (*Arachis* *monticola* Krapov. & Rigoni), and a cultivated peanut (*Arachis* *hypogaea* L.) [1,2]. Cultivated peanut is an allotetraploid derived from hybridization of two wild diploid species, *Arachis* *duranensis* Krapov. & W.C. Gregory (AA, 2n = 20) and *Arachis* *ipaensis* Krapov. & W.C. Gregory (BB, 2n = 20) [3]. Peanut is cultivated worldwide for its protein and edible oil content. It is grown in 100 countries producing 44 million metric tons from 26 million hectares area with China, India, and Nigeria as the top three producers accounting for about 61% of the world production based on FAOSTAT 2017 (http://www.fao.org/faostat/en/#home, accessed on 1 February 2022). Peanut seeds are known to be superior in oil (35 to 55%) and protein (20 to 30%) and contain crude fiber, vitamins, and minerals [4].

One of the main focuses of peanut breeding has been on kernel and pod traits, which are the main traits to determine the yield. Some of the critical characteristics to determine peanut quality are hundred seed weight, sound mature kernel (SMK), kernel weight percent (shelling percent), and loose shelled kernel (LSK). Studies have been conducted to identify QTLs for seed weight, seed size, pod length, pod weight, and other yield-related traits [5,6,7]. However, the genetic basis underlying pod- and kernel-related traits in the peanut remained largely unknown, which has hampered marker-assisted selection improvement of peanut.

Seed dormancy is a phenomenon of delaying germination by viable seed when exposed to favorable conditions. Preharvest sprouting (PHS) is a significant problem triggered by untimely rains before harvest. Such problems prevail in major crops such as rice, wheat, barley, and peanut, which reduces the overall market value of the crop [8,9,10]. In peanut, PHS in soil results in inferior seed quality and reduced yield. Around 10 to 20% of yield loss have been reported in peanut due to PHS [11]. Effects of plant hormones such as abscisic acid (ABA) and ethylene on dormancy have been studied in peanut [11,12]. *AhNCED1*, a gene encoding nine-cis-epoxycarotenoid dioxygenase regulates ABA biosynthesis and affects seed germination in peanut [12]. Studies have also been conducted to identify the inheritance of the dormancy trait and molecular markers associated with it [10,13]. Only a few genome-wide studies have been conducted using F2 mapping of populations to identify genomic regions associated with dormancy [10,14].

Genome-wide association (GWA) using a diverse panel is an efficient way to identify genomic regions for the trait being studied. Based on growth habit, morphological characteristics, inflorescence, and pod and seed characteristics, the cultivated peanut is classified into two main subspecies: *hypogea*, consisting of two botanical varieties (var. *hypogaea* and var. *hirsuta*), and *fastigiata*, consisting of four botanical varieties (var. *fastigiata*, var. *peruviana*, var. *aequatoriana*, and var. *vulgaris*) [15]. The U.S. mini-core collection consists of four different botanical varieties, namely, *fastigiata*, *peruviana*, *vulgaris*, and *hypogaea*. Spanish varieties (*fastigiata* and *vulgaris*) lack seed dormancy, while Virginia and runner types can maintain prolonged dormancy [11,13]. Here, we phenotyped a subset of the mini-core collection with wide genetic diversity for different seed germination and seed and pod traits and conducted whole-genome scanning using high-quality SNP arrays to identify genomic regions associated with seed development and germination.

## 2. Materials and Methods

### 2.1. Plant Materials and Phenotyping for Seed Quality and Germination

The diversity panel contained a total of 120 accessions mainly coming from the U.S. peanut mini-core collection (Table S1) [16]. This panel is comprised of six different botanical varieties, namely, *fastigiata*, *peruviana*, *vulgaris*, *aequatoriana*, *hirsute* and *hypogaea*. Each accession was planted in a two-row, 10-foot-long plot in Dawson, GA, USA, in 2010 using a randomized complete block design with three replications. Irrigation was applied before and after planting to keep the field adequately moist. Crop management for all tests was according to the best management practices for soil nutrients (N:P:K = 120 kg:26 kg:33 kg ha^−1^), and herbicides, insecticides, and fungicides were applied as required. A small peanut harvesting combine was used for harvesting, and the pods were dried to 10% of moisture for further evaluation of seed dormancy and germination, seed grading, and characteristics.

### 2.2. Assessment for Seed Dormancy and Germination

The procedure for seed germination testing has been documented in the previous publication [11]. The weight of 100 seeds was measured, and the seeds were treated with Trilex Star^®^ fungicide (Bayer CropScience, Research Triangle Park, NC, USA) before evaluating the accessions for seed germination. Two replications of 25 seeds were tested for seed germination by spreading them on a soaked germination paper towel and covering them with another paper towel. The temperature of the growth chamber was maintained at 30 °C for 12 h with 8 h of light and 20 °C for 12 h without any light, and the relative humidity was maintained at 85%. The number of germinated seeds was measured at 7, 14, and 21 days. A seed was declared dead if it showed no sign of germination and was rotten.

### 2.3. Evaluation for Seed Grading

The grading criteria, shelling percentage, sound mature kernels (SMKs), and loose shelled kernels (LSKs) were determined nondestructively using a prototype X-ray imaging device for grading peanuts (TOMRA, Leuven, Belgium). The LSK is defined as the peanut kernels in the sample that were shelled and separated from the shells or hulls during the harvesting process and is expressed as a percentage of the total sample weight. The percent KRS (kernels riding screen) are the kernels that, after shelling, will ride a 6.4 mm × 19 mm slotted screen and is expressed as a percentage of the total weight of clean in-shell pods. The percent KRS represents the peanuts that are of sufficient size to be sold as edible peanuts. SMKs are the KRS that have been sorted by hand and visually inspected for damage and the damaged/discolored kernels removed. The shelling percentage is the fraction of the peanut pods that are peanut kernels expressed as a percentage of the total weight of in-shell pods. The peanut sample pass under an X-ray energy source on a conveyor belt. A detector positioned below the belt detects the intensity of the X-ray spectrum transmitted through the peanut. A two-dimensional image is analyzed, classifying the particles in the image as foreign material, LSK, or in-shell peanuts. The density of each particle is proportional to the darkness of the gray-scale (darker is more dense), and the weight of the particle is estimated from the projected area and the density of the particle. Image analysis also estimates the weight of in-shell kernels with a minimum diameter greater than or equal to 6.4 mm, the weight of in-shell kernels smaller than 6.4 mm, total kernel weight, and the total hull weight.

### 2.4. SNP Genotyping Using SNP Array

**Figure 1:**
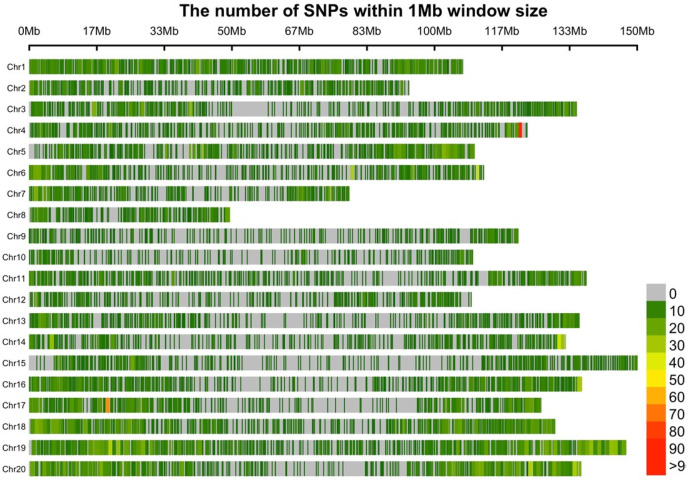
Distribution of SNP markers in the 20 chromosomes from genotyping of the association panel on the peanut genome. Horizontal axis represents physical distance along each chromosome. The color pattern on each chromosome indicates density of marker in the region of 1Mb window size.

Leaf tissue samples were collected for each accession, and DNA was isolated using the CTAB protocol. DNA quality and quantity were measured using NanoDrop-2000 (Thermo Scientific, Waltham, MA, USA). All the samples were genotyped on the Axiom_Arachis array [17,18] at GeneSeek (Lincoln, NE, USA). A total of 17,223 SNPs distributed on 20 chromosomes were polymorphic in the association panel (Figure 1).

### 2.5. Data Analysis

Correlation was determined between different seed grade, seed weight, germination rate and seed dormancy using the “PROC CORR” command of SAS software (SAS Institute Inc., SAS^®^9.2, Cary, NC, USA). Mean, range, and CV% were calculated using Excel, and graphs were drawn using Sigma Plot 13.0 (Systat Software, San Jose, CA, USA).

Population structure analysis was conducted using the principal component analysis (PCA) approach as described in the manual of GAPIT software [19] and software STRUCTURE v.2.3.4 [20]. Kinship analysis was conducted by following VanRaden’s method in GAPIT [21]. For structure analysis, the SNP data were used with the admixture model with five iterations of 50,000 burn-in and 50,000 Monte Carlo Markov Chain (MCMC) replications for k = 1 to k = 10. The ideal level of subpopulation was determined by calculating DeltaK using Structure Harvester [22].

All the phenotypic and genotypic data were imported in SVS software package (SNP & Variation Suite, Golden Helix, Bozeman, MT, USA), and genome-wide association analysis was performed as described in a previous publication [23]. A total of 13,382 SNPs were selected for GWAS after removing SNPs with a call rate <95% or a minor allele frequency (MAF) <0.05%. Linkage disequilibrium (LD) pruning at each LD testing and *r*^2^ = $\frac{D2}{pApBpapb\;}$ threshold of 0.2 were used for the determination of independent LD blocks [24]. A total of 1024 LD blocks were determined for this association panel. Principle component analysis was performed using independent SNPs identified in the LD block analysis. To correct for population stratification, GWAS analysis was performed using the efficient mixed-model association expedited (EMMAX) method [25] with the model of *Y* = *Xβ* + *Zu* + *e*. Here, Y is the vector of phenotype, *X* is incidence matrix for fixed effects, *β* is coefficient vector for fixed effects, *Z* is incidence matrix for random additive genetic effects, *u* is coefficients vector of random effect, and vector of random residuals is denoted by *e*. A Manhattan plot of the −log10 ^(*p* value)^ was also produced by the SVS software. Bonferroni-correction along with the number of independent SNPs were used to determine the threshold *p* value for genome-wide significance. Significant threshold was determined at *p* value at 0.05/1024 = 4.8 × 10^−5^ and −log10 ^(*p* value)^ = 4.31, while suggestive associating allowing one false-positive was determined at *p* value 1/1024 = 9.77 × 10^−4^ or −log10 ^(*p* value)^ = 3.01. Graphs were depicted using qqman R-package [26].

### 2.6. Candidate Gene Search

We used a recent sequence of tetraploid peanut cultivar (*Arachis hypogaea*, Tifrunner) genome to identify candidate genes [27] in a window of 1 Mb on both sides of a marker. Information related to gene position and annotation was download for *Arachis hypogaea* v1.0 gene models from https://peanutbase.org/gbrowse_peanut1.0 (access on 7 July 2020) and extracted using Python script.

## 3. Results

### 3.1. Correlation and Distribution of Dormancy and Seed Quality Traits

As expected, the germination rate at different time points had a strong negative correlation to seed dormancy. Seed weight negatively correlated to germination and positively correlated to dormancy. Similarly, LSK and shelling percent positively correlated with germination and negatively correlated with dormancy. Seed weight also showed a negative correlation to LSK and SMK. Finally, shelling percent had a negative correlation with SMK (Table 1).

Germination percent ranged from 0 to 100% at different time points with an average of 50.6%, 54.6%, and 57.1% at 7, 14, and 21 days after treatment, respectively. Similarly, seed dormancy ranged from 0 to 100%, with an average of 41.5%. The coefficients of variation (CV) suggest that shelling percent is the least variable trait, while seed weight, LSK, and SMK are highly variable traits among the mini-core accessions (Table 2).

### 3.2. Distribution of SNP Markers, Linkage Disequilibrium, and Population Structure

**Figure 2:**
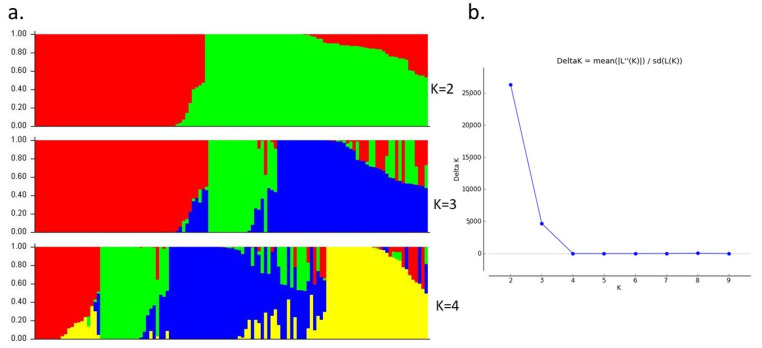
Population structure analysis using membership probability (Q-values) for 120 peanut genotypes. (**a**) Bar plot for different possibility of subpopulation K = 2 to 4 was created with each color representing one cluster. (**b**) Delta K (ΔK) calculated between K = 2 to K = 9 using information from STRUCTURE analysis indicates that there are two major subpopulations (K = 2).

The association panel consisting of 120 accessions was genotyped using the Axiom_Arachis array. A total of 17,223 high-quality SNPs were found to be polymorphic, which were distributed in twenty chromosomes covering 2371.61 Mb region of the genome with 137.7 kb of the average distance between two SNPs (Figure 1). The mean distance between two SNPs for each chromosome ranged from 87.14 kb (Chr1) to 291.36 kb (Chr10). A total of 1024 LD blocks were identified through linkage disequilibrium pruning using a window size of 50 SNPs and *r*^2^ of 0.2 in the SVS software. Population analysis was conducted by structure analysis using high-quality SNPs (Figure 2a). The Delta K (ΔK) graph, which only peaked at K = 2 (Figure 2b), suggests there are two major subgroups. One group consists of 68 genotypes of the diversity panel, while the second group consists of 52 genotypes.

### 3.3. Genomic Regions Associated with Quantitative Traits and Perusal for Candidate Genes

**Figure 3:**
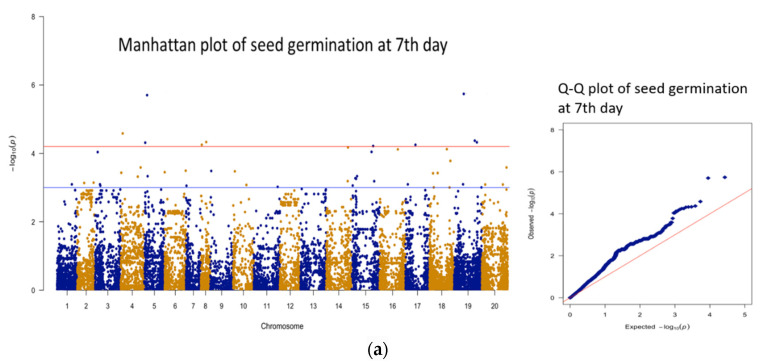

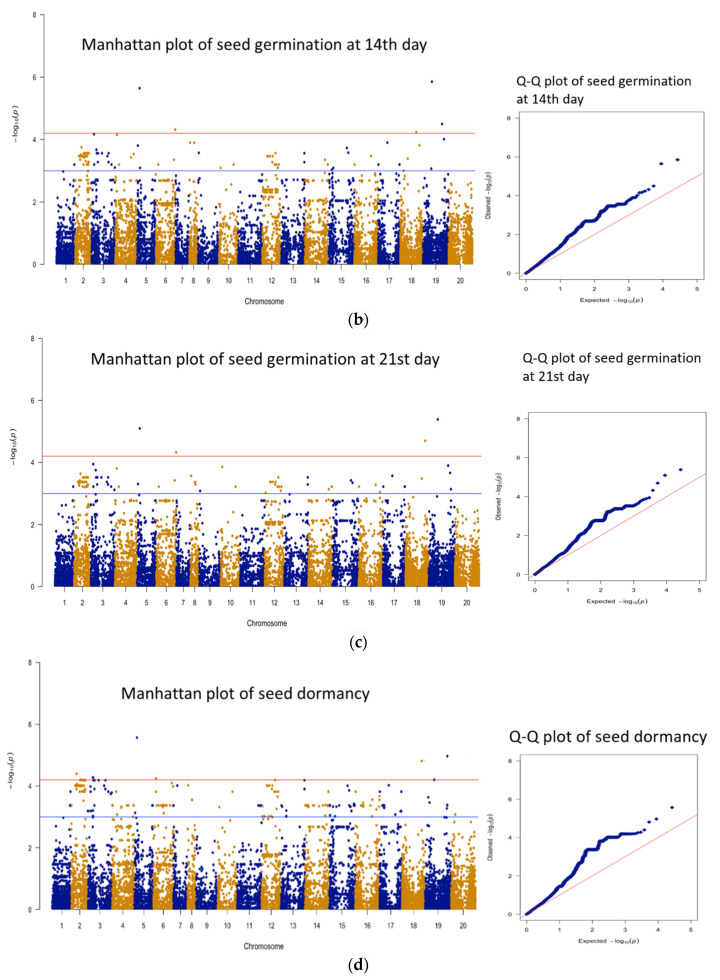
Manhattan and Q−Q plots for seed germination rate at the 7th (**a**), 14th (**b**), and 21 days (**c**), and seed dormancy (**d**). Significant threshold is marked by a red line, and suggestive threshold is marked by a blue line in the Manhattan plot.

A total of seven, four, four, and four SNPs were significantly associated with germination at 7, 14, and 21 days and seed dormancy, respectively (Table 3, Figure 3). Furthermore, SNP markers with suggestive association with germination at 7, 14, and 21 days and seed dormancy were found on a total of 18, 17, 16, and 17 chromosomes, respectively (Figure 3). Common SNPs were identified, showing significant association with germination at different time points and seed dormancy. SNP marker AX-176814819 (Chr A05) was significantly associated with germination at different time points and seed dormancy, which explains 18.3 to 20.5% of phenotypic variance, whereas AX-177644204 (Chr B09) was significantly associated with germination at 7, 14, and 21 days, which explains 19.4 to 21% of phenotypic variance (PV) (Table 4). In addition, SNP AX-177643736 (Chr B09, explaining around 16% PV) showed a significant association for germination at 7 and 14 days; SNP AX-176799628 (Chr A06, 15%) showed a significant association for germination at 14 and 21 days; and SNP AX-147258769 (Chr B08, 17%) showed significant association for germination at 21 days and seed dormancy (Table 4).

**Figure 4:**
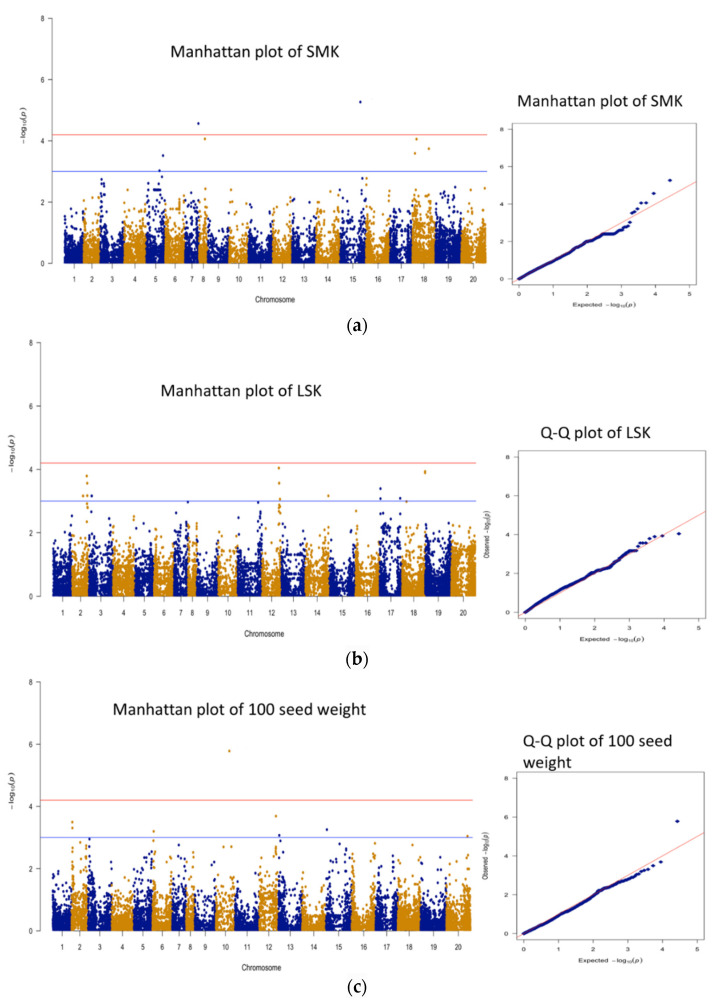

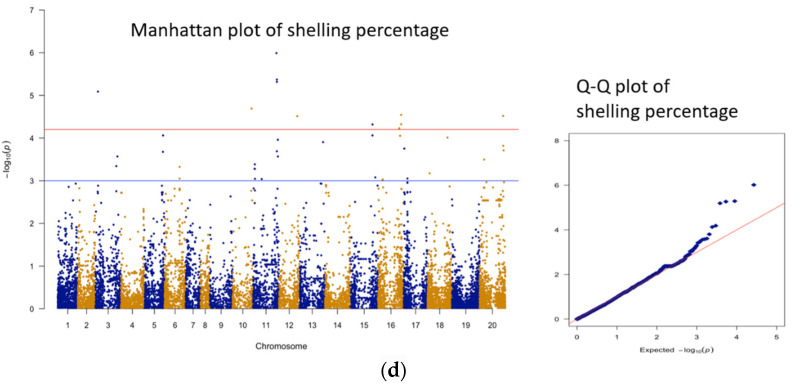
Manhattan and Q−Q plots for seed germination at SMK (**a**), LSK (**b**), 100 seed weight (**c**), and shelling percent (**d**). Significant threshold is marked by a red line, and suggestive threshold is marked by a blue line in the Manhattan plot.

Pod and seed quality are essential components to determine the overall price of peanut. A total of 13 (significantly associated) and 69 (suggestive) genomic regions were found for seed characteristics, i.e., SMK, LSK, shelling percent, and 100 seed weight (Table 3, Figure 4). Markers AX-176823847 (Chr B05) and AX-176810056 (Chr A07) were found significantly associated with SMK, explaining 17.0 and 14.7% phenotypic variation, respectively (Figure 4, Table 5). A total of 10 significant markers were associated with shelling percent, which explains 13.9 to 19.4% of phenotypic variation (Figure 4, Table 5). For seed weight, marker AX-177638040 (Chr A10) was significantly associated and explains 20.8% of phenotypic variation (Table 5). No significant marker was found for LSK.

Genes that play a putative role in seed development or germination were searched in the vicinity of the significant markers. A total of 832 genes were identified in the vicinity of 11 significantly associated SNPs (excluding redundant associated SNPs) to seed germination and dormancy (Figure 3, Table 4). Marker AX-176814819 on chromosome A05 was found to be associated at all readings of germination (7, 14, and 21 days) and seed dormancy. There are 102 putative genes presenting in the 1 Mb region of this marker, many of which that have functions in the hormonal biosynthesis pathway, transcription factors, lipid metabolism, and other biochemical pathways that can affect fertility, growth, and development of plants. Similarly, there are 102, 21, and 715 (excluding redundant genes from common regions) genes in the vicinity of 2, 1, and 10 significantly associated genomic regions for SMK, seed weight, and shelling percent (Figure 4, Table 5).

## 4. Discussion

QTLs studies use biparental populations with few recombination opportunities and are restricted to genetic variation between two parents. In contrast, a GWAS study with a diversity panel can provide higher resolution and identify more genomic regions that contribute to the quantitative traits. In the current GWAS study, we did corroborate previously known genomic regions for seed, pod, and germination traits. We identified some novel genomic regions that have never been reported for these traits. In total, we identified 277 SNPs associated (excluding redundant associated SNPs) (Table 3) with either seed germination or dormancy, 8 SNPs associated with SMK, 17 SNPs associated with LSK, 8 SNPs associated with 100 seed weight, and 49 SNPs associated with shelling percent. Some of these genomic regions have been identified in previous studies, but many are reported for the first time in this GWAS study.

Many traits studied here had a significant correlation, suggesting a good chance of co-localizing these quantitative traits on the same chromosome. SMK and shelling percent had a strong correlation of −0.4, and we found that SNPs strongly associated with these traits were co-localized on chromosomes A05, B05, and B08 (Supplemental Table S2). Shelling percent and number of seeds germinated at seven days were strongly correlated (r = 0.42) (Table 1), and SNPs linked to these traits were present in near proximity on chromosomes A03, B01, B05, B08, and B10 (Supplemental Table S2). Weight for 100 seed weight and number of seeds germinated at seven days were strongly correlated (r = −0.38), and SNPs linked to these traits were co-localized on chromosomes A06, A10, B05, and B10 (Supplemental Table S2). We also found many regions co-localized on the same chromosome associated with multiple traits. For example, a 3 Mb region on Chr. B02 was associated with LSK, shelling percent, seed dormancy, and 100 seed weight. Similarly, a 4.8 Mb region on Chr. B05 and a 7.5 Mb region on Chr. B08 had an association with SMK, shelling percent, and seed germination (Supplemental Table S2). Previous studies have identified co-localized regions on chromosome different seed trait and pod traits, but this is the first time we report co-localized regions between seed and pod traits with seed germination and dormancy using a diversity panel [28,29,30,31]. These regions can expedite the peanut breeding program, as the targeted genomic region can improve multiple traits.

It was also discerned that SNPs associated with a trait were present on both homoeologous chromosomes, thus suggesting similar genomic regions of A and B subgenomes contribute to these traits. For example, we identified SNPs associated with 100 seed weight on chromosomes A02, B02, A10, and B10. Similarly, we identified SNPs related to SMK on chromosomes A05, B05, A08, and B08. For shelling percent, we found associated SNPs on chromosomes A03, B03, A05, B05, A06, B06, A10, and B10.

We identified an SNP on chromosome A05 to be associated with seed germination as well as seed dormancy, and it explains about 20% of phenotypic variation. Previous research work has identified a major QTL on chromosome A05 for seed dormancy [10]. Another work using QTL-seq approach found two major QTLs on chromosome B05 and A09 controlling fresh seed dormancy in peanut [14]. Interestingly, in our study, we found SNPs on homoeologous chromosomes A05 and B09 were strongly associated with seed germination and dormancy in peanut. A total of 102 and 16 genes affecting seed germination were identified in the vicinity of SNPs of these chromosomes, respectively. Arahy.B8HL1K on chromosome B09 encodes ascorbate peroxidase, which maintains the level of reactive oxygen species (ROS) and regulates timings of seed germination and preservation of seed dormancy [32,33]. Arahy.KDM1H7 on A05 encodes acyltransferase enzymes, important in anther development, maintaining fertility, and seed germination [34,35]. Arahy.K0UY8K and Arahy.JH8TMD on chromosome A05 encode chalcone synthase, which is the first enzyme in flavonoid biosynthesis, producing proanthocyanidins (PAs) [36]. Presence of proanthocyanidins in seed coats have contributed to seed dormancy in many plant species [36,37,38]. Another two genes, Arahy.MPKI4V and Arahy.NAXY0P, on chromosome A05 encode Cytochrome P450, which is responsible for regulating many plant hormones and thus plays an important role in seed germination. Cytochrome P450 (CYP707A) in Arabidopsis is vital for seed germination and regulation of ABA (abscisic acid) concentration in the plant tissue [39].

We also identified genomic regions on chromosomes A02, A04, A06, A08, and B08 to be strongly associated with seed dormancy and early seed germination. Such regions might also be vital for peanut breeding program for seed dormancy, which have been missed in QTL studies contrived on fewer biotypes of the peanut core collection. These regions also contain many crucial genes for seed dormancy, for example, in vicinity of associated region of Chr. A04, we found multiple copies of chalcone synthase, cytochrome P450 (Arahy.10MYPD), WRKY transcription factor [40,41], different forms of transcriptional factor, gene for gibberellin biosynthesis (Arahy.3B8AB8), and polygalacturonase (Arahy.08SQ5V) [42,43].

For SMK, we identified two strongly associated genomic regions on chromosomes A07 and B05. A QTL study using a RIL population found QTL for TSMK (total sound mature kernel) on chromosomes A07 and A05 (homoeologous of B05). A05 and B05 chromosomes are also known to have QTLs for seed weight, seed size, and pod size [29,31]. Further, QTL for TSMK was also found on A08, where we identified SNPs that were suggestively linked to SMK [44]. A total of 73 and 29 genes were identified around SNPs of A07 and B05, respectively, which were strongly associated with SMK. Among them, there were transcription factors, protein kinesis, fatty acid hydroxylase, cytochrome P450, Ulp1 protease, galactosyltransferase, pentatricopeptide repeat, NAD(H) kinase, and other genes that have roles in cell elongation, cytoskeleton formation, lipid synthesis, metabolic control, cell-cycle progression, oil content, maintaining ROS accumulation during development, and seed and embryo development [45,46,47,48,49,50]. For seed weight, we identified a significant association with a marker on Chr. A10. A SSR marker, GM2531, on Chr A10 was found to be strongly associated with seed weight [51,52]. A QTL on B10 (homoeologous of A10) was identified for 100 seed weight, 100 pod weight, pod area, and yield [29]. We also identified SNPs on chromosomes A02, A06, B05, and B10 associated with 100 seed weight. QTLs have been identified on these chromosomes for seed size, seed weight, pod weight, pod length, pod width, and yield [7,29,31]. A total of 21 putative genes were identified around the strongly associated SNP on Chr. A10, which consists of WRKY transcription factor, tetratricopeptide repeat, ABC transporter, and other genes associated with seed development and seed size [53,54]. A total of 10 SNPs on chromosomes A03, A10, B01, B02, B05, B06, and B10 were found to be significantly associated with shelling percent. A GWAS using 300 genotypes of peanut and 154 SSR markers identified GM1899 and GM2531 on chromosomes B01 and A10, respectively, to be associated with shelling percent. We identified three SNPs on Chr. B01 and one SNP on Chr. A10 that were strongly associated with shelling percent [51,52]. Interestingly, SSR marker GM2531 on Chr. A10 was associated with seed weight, and shelling percent suggests a hypothesis that genomic regions for these traits are co-localized, and we also found SNPs associated with shelling percent and seed weight that were only 21.8 Mb apart from each other. A major QTL was identified for shelling percent on Chr. B02 in recent QTL-seq study using RIL population developed from the cross Yuanza 9102 × Xuzhou 68–4. A study using the RIL population was conducted to identify QTLs for shelling percent and found stable QTLs on chromosomes B02, B05, and B10 [55]. Furthermore, QTLs for correlated traits to shelling percent such as seed length and seed width were identified on A03 [56]. Thus, regions identified in this GWAS study for shelling percent have been previously reported, providing strong support to the findings in this study. A total of 715 genes were identified in the vicinity of 10 SNPs associated with shelling percent, which should be further evaluated to understand the genetic mechanism.

## 5. Conclusions

In summary, we identified previously known and novel genomic regions associated with seed quality traits and germination. Such regions can now be used for enhancing the peanut breeding program. Many candidate genes also identified in the vicinity of the associated marker warrant further research to identify their roles in seed development and germination.

## Figures and Tables

**Table 1:** Estimating correlations between seed trait using Pearson Correlation Coefficient.

	7th Day	14th Day	21st Day	Seed Dormancy	Seed Weight	LSK	SMK
14th day	0.99 *						
21st day	0.98 *	0.99 *					
Seed dormancy	−0.97 *	−0.98 *	−0.99 *				
Seed weight	−0.38 *	−0.33 *	−0.34 *	0.35 *			
LSK	0.43 *	0.41 *	0.41 *	−0.4 *	−0.38 *		
SMK	0.02	−0.01	−0.01	0	−0.6	0.23	
Shelling %	0.42 *	0.45 *	0.45 *	−0.43 *	0.1	0.24	−0.4 *

7th, 14th, and 21st represent germination rate at 7th, 14th, and 21st days, respectively. “*” denotes a significant correlation at *p* < 0.0001. Weight of hundred seeds (seed weight), sound mature kernel (SMK), shelling percent, and loose shelled kernel (LSK).

**Table 2:** Variation for different seed grading traits in diversity panel.

Variable	Min	Max	Mean	Std Dev	CV
SMK	2.7	45.8	12.3	7.28	59
LSK	0.26	9.57	2.59	2.09	81
seed weight	27.55	95.68	48.49	12.87	27
shelling percent	64.11	78.08	72.79	2.62	3.6

Weight of hundred seeds (seed weight), sound mature kernel (SMK), shelling percent, and loose shelled kernel (LSK).

**Table 3:** Summary of the number of associated SNP for seed germination, dormancy, and seed grading.

Traits	Number of Significant Associations	Number of Suggestive Associations
Germination rate at 7 days	7	38
Germination rate at 14 days	4	92
Germination rate at 21 days	4	87
Seed Dormancy	4	246
Gemination and Dormancy *	11	266
SMK	2	6
shelling percent	10	39
LSK	0	17
seed weight	1	7
Total	32	532

* The values represent total number of associated SNPs after removing the redundant associated SNPs. Weight of hundred seeds (seed weight), sound mature kernel (SMK), shelling percent, and loose shelled kernel (LSK).

**Table 4:** Significant markers associated with seed germination at a different time intervals and seed dormancy.

#	Trait	SNP Marker	Chr.	Position on Chr.	−log10 ^(*p* value)^	PVE (%)	# of Genes in Vicinity
1	7 days *	AX-177644204	B09	51563617	5.74	20.6	16
2	7 days	AX-176814819	A05	17060254	5.70	20.5	102
3	7 days	AX-176816015	A04	11035231	4.58	16.4	86
4	7 days	AX-177643736	B09	109630028	4.37	15.6	30
5	7 days	AX-176803643	A08	28348986	4.33	15.5	142
6	7 days	AX-177642270	B09	120713638	4.32	15.5	41
7	7 days	AX-147221650	A05	7266706	4.31	15.4	126
1	14 days	AX-177644204	B09	51563617	5.85	21	16
2	14 days	AX-176814819	A05	17060254	5.64	20.3	102
3	14 days	AX-177643736	B09	109630028	4.50	16.1	30
4	14 days	AX-176799628	A06	110978824	4.32	15.4	155
1	21 days	AX-177644204	B09	51563617	5.39	19.4	16
2	21 days	AX-176814819	A05	17060254	5.10	18.3	102
3	21 days	AX-147258769	B08	110623990	4.70	16.8	50
4	21 days	AX-176799628	A06	110978824	4.32	15.5	155
1	Seed Dormancy	AX-176814819	A05	17060254	5.57	20	102
2	Seed Dormancy	AX-177638083	B09	125395376	4.97	17.8	61
3	Seed Dormancy	AX-147258769	B08	110623990	4.81	17.3	50
4	Seed Dormancy	AX-176802837	A02	28578269	4.40	15.7	23

* Germination rate recorded at 7, 14, and 21 Days. Chr. is chromosome.

**Table 5:** Significant markers associated with seed grading traits.

#	Trait	SNP Marker	Chr.	Position on Chr.	−log10 ^(*p* value)^	PVE (%)	# of Genes in Vicinity
1	SMK	AX-176823847	B05	118253381	5.27	17.0	29
2	SMK	AX-176810056	A07	75161338	4.57	14.7	73
1	seed wt	AX-177638040	A10	76559318	5.78	20.8	21
1	shelling %	AX-176811900	B01	123973617	5.99	19.4	113
2	shelling %	AX-176806228	B01	126274566	5.37	17.4	121
3	shelling %	AX-176807776	B01	126179070	5.32	17.2	121
4	shelling %	AX-147216060	A03	13426004	5.09	16.5	98
5	shelling %	AX-147236668	A10	101148536	4.69	15.2	95
6	shelling %	AX-176808276	B06	122427781	4.54	14.7	74
7	shelling %	AX-177640468	B10	124893402	4.52	14.6	49
8	shelling %	AX-176813093	B02	97213496	4.51	14.6	79
9	shelling %	AX-176793701	B06	121887242	4.33	13.9	71
10	shelling %	AX-176802081	B05	120108986	4.32	13.9	68

Weight of hundred seeds (seed weight), sound mature kernel (SMK), shelling percent, and loose shelled kernel (LSK). Chr. is chromosome.

## Data Availability

Data is contained within the article and Supplementary Material.

## Supplementary Materials

The following supporting information can be downloaded at: https://www.mdpi.com/article/10.3390/plants11070837/s1, Table S1: Information of diversity panel consisting of 120 accessions used in this GWAS study; Table S2: Co-localizing genomic regions for seed quality traits, seed germination, and seed dormancy discovered in this GWAS study.

## Abbreviations

SNP—single nucleotide polymorphism, GWAS—genome-wide association study, LD—linkage disequilibrium, MAF—minor allele frequency, PVE—phenotypic variation explained, QTL—quantitative trait loci, Chr.—chromosome, SMK—sound mature kernel, LSK—loose shelled kernels, KRS—kernels riding screen.
