# Supplementary material for: Insights into the Genomic Architecture of Seed and Pod Quality Traits in the U.S. Peanut Mini-Core Diversity Panel
Source: Plants (Basel). 2022 Mar 22;11(7):837. doi: 10.3390/plants11070837 (PMC9003526; doi:10.3390/plants11070837)
Supplement: Supplementary file 1 [file plants-11-00837-s001.zip › plants-1629847-supplementary.pdf]

Table S1 Information of diversity panel consisting of 120 accessions used in this GWAS study

#	PI no.	Origin	Botanical variety
1	Grif12545	Ecuador	*aequatoriana*
2	Grif14051	Guatemala	*aequatoriana*
3	PI152146	Uruguay	*fastigiata*
4	PI155107	Uruguay	*vulgaris*
5	PI157542	China	*vulgaris*
6	PI158854	China	*fastigiata*
7	PI159786	Senegal	*hypogaea*
8	PI162655	Uruguay	*hypogaea*
9	PI162857	Sudan	*hypogaea*
10	PI196622	Cote D'Ivoire	*hypogaea*
11	PI196635	Madagascar	*hypogaea*
12	PI200441	Japan	*fastigiata*
13	PI240560	South Africa	*hypogaea*
14	PI259617	Cuba	*fastigiata*
15	PI259658	Cuba	*hypogaea*
16	PI259748	Peru	*hypogaea*
17	PI259836	Malawi	*fastigiata*
18	PI259851	Malawi	*hypogaea*
19	PI262038	Brazil	*fastigiata*
20	PI268586	Zambia	*hypogaea*
21	PI268696	South Africa	*hypogaea*
22	PI268755	Zambia	*hypogaea*
23	PI268806	Zambia	*hypogaea*
24	PI268868	Sudan	*hypogaea*
25	PI268996	Zambia	*hypogaea*
26	PI270786	Zambia	*hypogaea*
27	PI270905	Zambia	*hypogaea*
28	PI270907	Zambia	*hypogaea*
29	PI270998	Zambia	*vulgaris*
30	PI271019	Zambia	*vulgaris*
31	PI288146	India	*vulgaris*
32	PI288210	India	*vulgaris*
33	PI290536	India	*hypogaea*
34	PI290560	India	*vulgaris*

35	PI290566	India	*fastigiata*
36	PI290594	India	*hypogaea*
37	PI290620	Argentina	*fastigiata*
38	PI292950	South Africa	*hypogaea*
39	PI295250	Israel	*hypogaea*
40	PI295309	Israel	*hypogaea*
41	PI295730	India	*fastigiata*
42	PI296550	Israel	*hypogaea*
43	PI296558	Israel	*hypogaea*
44	PI298854	South Africa	*hypogaea*
45	PI313129	Taiwan	*fastigiata*
46	PI319768	Israel	*hypogaea*
47	PI323268	Pakistan	*hypogaea*
48	PI325943	Venezuela	*hypogaea*
49	PI331297	Argentina	*hypogaea*
50	PI331314	Argentina	*hypogaea*
51	PI337293	Brazil	*hypogaea*
52	PI337399	Morocco	*hypogaea*
53	PI337406	Paraguay	*fastigiata*
54	PI338338	Venezuela	*peruviana*
55	PI339960	Argentina	*fastigiata*
56	PI343384	Israel	*hypogaea*
57	PI343398	Israel	*fastigiata*
58	PI355268	Mexico	*hypogaea*
59	PI355271	Mexico	*hypogaea*
60	PI356004	Argentina	*fastigiata*
61	PI370331	Israel	*hypogaea*
62	PI371521	Israel	*hypogaea*
63	PI372271	Unknown	*hypogaea*
64	PI372305	Nigeria	*hypogaea*
65	PI390428	Ecuador	*hypogaea*
66	PI399581	Nigeria	*hypogaea*
67	PI403813	Argentina	*vulgaris*
68	PI407667	Thailand	*vulgaris*
69	PI429420	Zimbabwe	*fastigiata*
70	PI442768	Zimbabwe	*hypogaea*

71	PI461427	China	*hypogaea*
72	PI461434	China	*hypogaea*
73	PI468250	Bolivia	*hypogaea*
74	PI471952	Zimbabwe	*hypogaea*
75	PI471954	Zimbabwe	*fastigiata*
76	PI475863	Bolivia	*fastigiata*
77	PI475918	Bolivia	*fastigiata*
78	PI476025	Peru	*fastigiata*
79	PI476432	Nigeria	*hypogaea*
80	PI476636	Nigeria	*hypogaea*
81	PI478819	India	*vulgaris*
82	PI478850	Uganda	*fastigiata*
83	PI481795	Mozambique	*hypogaea*
84	PI482120	Zimbabwe	*hypogaea*
85	PI482189	Zimbabwe	*fastigiata*
86	PI493329	Argentina	*fastigiata*
87	PI493356	Argentina	*fastigiata*
88	PI493547	Argentina	*fastigiata*
89	PI493581	Argentina	*fastigiata*
90	PI493631	Argentina	*fastigiata*
91	PI493693	Argentina	*fastigiata*
92	PI493717	Argentina	*fastigiata*
93	PI493729	Argentina	*fastigiata*
94	PI493880	Argentina	*fastigiata*
95	PI493938	Argentina	*fastigiata*
96	PI494018	Argentina	*vulgaris*
97	PI494034	Argentina	*vulgaris*
98	PI494795	Zambia	*hypogaea*
99	PI496401	Burkina Faso	*hypogaea*
100	PI496448	Burkina Faso	*hypogaea*
101	PI497318	Bolivia	*hypogaea*
102	PI497395	Bolivia	*hypogaea*
103	PI497517	Brazil	*fastigiata*
104	PI497639	Ecuador	*fastigiata*
105	PI497648	Ecuador	*fastigiata*
106	PI501272	Peru	*hypogeae*

107	PI502040	Peru	*fastigiata*
108	PI502111	Peru	*peruviana*
109	PI502120	Peru	*peruviana*
110	PI504614	Colombia	*hypogaea*
111	PI576613	Mexico	*hirsuta*
112	PI576614	Mexico	*hirsuta*
113	PI576634	Mexico	*hirsuta*
114	PI576636	Mexico	*hirsuta*
115	PI576637	Mexico	*hirsuta*
116	PI648241	Ecuador	*hirsuta*
117	PI648242	Ecuador	*aequatoriana*
118	PI648245	Ecuador	*aequatoriana*
119	PI648249	Ecuador	*aequatoriana*
120	PI648250	Ecuador	*aequatoriana*

Table S2 Co-localizing genomic regions for seed quality traits, seed germination, and seed dormancy discovered in this GWAS study.

Trait	SNP marker	Chr.	position	$-\log10^{(P\text{-Value})}$	PVE (%)
SMK	AX-147223416	A05	97286880	3.52	11.14
shelling %	AX-176818835	A05	100814719	3.68	11.69
SMK	AX-176823847	B05	118253381	5.27	17.07
shelling %	AX-176813166	B05	119858337	4.06	13.01
shelling %	AX-177643293	B08	11599042	3.17	9.94
SMK	AX-177642165	B08	14742332	3.59	11.39
shelling %	AX-147216060	A03	13426004	5.09	16.48
7days	AX-176801636	A03	14091817	4.04	14.39
shelling %	AX-176811900	B01	123973617	5.99	19.44
7days	AX-176823831	B01	125913979	3.02	10.49
shelling %	AX-147239086	B01	126117760	3.69	11.74
shelling %	AX-176802081	B05	120108986	4.32	13.89
7days	AX-176797808	B05	121063906	3.19	11.13
shelling %	AX-147258677	B08	106271132	4.01	12.84
7days	AX-147258769	B08	110623990	3.78	13.41
shelling %	AX-176823311	B10	127323301	3.71	11.81
7days	AX-147264901	B10	129930580	3.59	12.68
7days	AX-176811574	A10	70323232	3.08	10.71
seed wt	AX-177638040	A10	76559318	5.78	20.79
7days	AX-147224061	A06	51298	3.45	12.15
seed wt	AX-176801078	A06	6527694	3.20	11.17
7days	AX-176822445	B05	7631930	3.06	10.64
seed wt	AX-176817345	B05	8733975	3.25	11.39
7days	AX-177639567	B10	110673150	3.09	10.76
seed wt	AX-177639117	B10	120152356	3.04	10.57
shelling %	AX-147216060	A03	13426004	5.09	16.48
7, 14,21, Seed Dormancy	AX-176801636	A03	14091817	4.17	14.88
14, 21, Seed Dormancy	AX-176807956	A03	14799828	3.20	11.17
LSK	AX-176820794	A03	15685730	3.16	9.9
Seed Dormancy	AX-176799002	A03	15708945	3.19	11.15
LSK	AX-176821054	A03	16832251	3.16	9.9
SMK	AX-147223416	A05	97286880	3.52	11.14
shelling %	AX-176818835	A05	100814719	3.68	11.69
shelling %	AX-147223558	A05	101618480	4.06	13.01
Seed Dormancy	AX-176819528	A05	102860702	3.37	11.84
Seed Dormancy	AX-176804849	A05	106482015	3.37	11.84
seed wt	AX-177638040	A10	76559318	5.78	20.79
14, 21, Seed Dormancy	AX-177641629	A10	83734285	3.20	11.17

shelling %	AX-147236668	A10	101148536	4.69	15.15
14, 21, Seed Dormancy	AX-176805268	B02	94348554	3.82	13.55
LSK	AX-176811899	B02	95026708	4.04	12.95
seed wt	AX-176820704	B02	95335297	3.69	13.06
LSK	AX-176822571	B02	96680636	3.56	11.29
LSK	AX-176823190	B02	96680828	3.57	11.3
shelling %	AX-176813093	B02	97213496	4.51	14.55
7, 14,21, Seed Dormancy	AX-176809013	B05	115522414	3.86	13.69
Seed Dormancy	AX-176797150	B05	116237196	3.37	11.84
SMK	AX-176823847	B05	118253381	5.27	17.07
shelling %	AX-176813166	B05	119858337	4.06	13.01
shelling %	AX-176802081	B05	120108986	4.32	13.89
14, 21, Seed Dormancy	AX-176818622	B07	2173393	3.82	13.55
shelling %	AX-177639525	B07	2839129	3.75	11.95
LSK	AX-147254688	B07	4382301	3.39	10.7
LSK	AX-147254691	B07	4382872	3.08	9.6
shelling %	AX-177643293	B08	11599042	3.17	9.94
SMK	AX-177642165	B08	14742332	3.59	11.39
7days	AX-176821555	B08	19124117	3.42	12.02

7th, 14th, and 21st represent germination rate at 7th, 14th, and 21st day.

Weight of hundred seeds (seed wt), Sound Mature Kernel (SMK), shelling percent, and Loose Shelled Kernel (LSK)
